# A routine tool with far-reaching influence: Australian midwives’ views on the use of ultrasound during pregnancy

**DOI:** 10.1186/s12884-015-0632-y

**Published:** 2015-08-27

**Authors:** Kristina Edvardsson, Ingrid Mogren, Ann Lalos, Margareta Persson, Rhonda Small

**Affiliations:** Department of Clinical Sciences, Obstetrics and Gynecology, Umeå University, SE 901 87 Umeå, Sweden; Judith Lumley Centre, La Trobe University, 215 Franklin Street, Melbourne, Vic 3000 Australia; Department of Nursing, Umeå University, Umeå, SE 901 87 Sweden

**Keywords:** Midwives, Ethics, Fetus, Maternal rights, Obstetric ultrasound, Obstetrics, Pregnant women, Prenatal diagnosis, Qualitative studies

## Abstract

**Background:**

Ultrasound is a tool of increasing importance in maternity care. Midwives have a central position in the care of pregnant women. However, studies regarding their experiences of the use of ultrasound in this context are limited. The purpose of this study was to explore Australian midwives’ experiences and views of the role of obstetric ultrasound particularly in relation to clinical management of complicated pregnancy, and situations where maternal and fetal health interests conflict.

**Methods:**

A qualitative study was undertaken in Victoria, Australia in 2012, based on six focus group discussions with midwives (*n* = 37) working in antenatal and intrapartum care, as part of the CROss-Country Ultrasound Study (CROCUS). Data were analysed using qualitative content analysis.

**Results:**

One overarching theme emerged from the analysis: *Obstetric ultrasound – a routine tool with far-reaching influence,* and it was built on three categories. First, the category*‘Experiencing pros and cons of ultrasound’* highlighted that ultrasound was seen as having many advantages; however, it was also seen as contributing to increased medicalisation of pregnancy, to complex and sometimes uncertain decision-making and to parental anxiety. Second, *‘Viewing ultrasound as a normalised and unquestioned examination’* illuminated how the use of ultrasound has become normalised and unquestioned in health care and in wider society. Midwives were concerned that this impacts negatively on informed consent processes, and at a societal level, to threaten acceptance of human variation and disability. Third, ‘*Reflecting on the fetus as a person in relation to the pregnant woman*’ described views on that ultrasound has led to increased ‘personification’ of the fetus, and that women often put fetal health interests ahead of their own.

**Conclusions:**

The results reflect the significant influence ultrasound has had in maternity care and highlights ethical and professional challenges that midwives face in their daily working lives concerning its use. Further discussion about the use of ultrasound is needed, both among health professionals and in the community, in order to protect women’s rights to informed decision-making and autonomy in pregnancy and childbirth and to curb unnecessary medicalisation of pregnancy. Midwives’ experiences and views play an essential role in such discussions.

## Background

The use of ultrasound in pregnancy for screening, diagnosis and pregnancy management purposes is established practice in most developed countries, and is increasingly established also in developing countries [1]. Midwives play an important role in the provision of sexual, reproductive, maternal and newborn health care, and can provide an estimated 87 % of the essential care needed for women and newborns, according to the World Health Organization. Despite differences in spheres of practice in different countries, there can be little doubt that midwives play an important role in the global prevention of maternal and newborn mortality and morbidity [2]. The extent to which the use of ultrasound is incorporated in midwifery practice varies widely across countries. For example in Sweden, it is commonly specially trained midwives who perform routine ultrasound screening in obstetrics departments [3]. Midwife use of ultrasound in low income-countries has been shown to be positive, improving accuracy of diagnosis, assisting midwives in clinical decision-making and pregnancy management, and also lessening the workload of specialists [4–6]. Although the use of ultrasound is not detailed as a component of midwives’ scope of practice in Australia, it has become an increasingly integral part of overall pregnancy management, particularly for midwives working in fetal medicine units and with women experiencing complicated pregnancies.

Pregnant women in Australia have a range of options for models of maternity care [7]. Nearly all undergo at least one ultrasound examination during pregnancy, with the vast majority undergoing the second trimester routine ultrasound examination [8]. Ultrasound is known to be highly valued by pregnant women; having an ultrasound examination is seen as an opportunity for getting reassurance that everything is fine with the pregnancy, getting confirmation that the pregnancy is real, and ‘meeting the baby’ [3, 9]. However, research has shown that women are often insufficiently informed about the purpose of the routine ultrasound examination in pregnancy, which not surprisingly can lead to distress and anxiety if abnormalities are detected. Obstetric ultrasound examinations can also provide false reassurance if the limitations of the scan are not well understood [9]. Many factors can affect the accuracy, including gestation, fetal position, BMI, the quality of the equipment and the operator’s expertise and skills [10]. Thus, a negative result will not always result in the birth of a healthy infant.

Ultrasound is undoubtedly a tool of established and increasing importance in obstetric management. Midwives have a central position in maternity care, and are commonly the professional group working closest to the pregnant woman in pregnancy, labour and childbirth. Because ultrasound is used in the care of nearly every pregnant woman, it is also central to midwives’ everyday practice, and particularly when ultrasound findings have an impact on the care provided. However, studies investigating midwives’ experiences and views of using ultrasound in pregnancy management are limited.

The overall purpose of this study was to explore Australian midwives’ experiences and views of the role of obstetric ultrasound particularly in relation to clinical management of complicated pregnancy, and in situations where maternal and fetal health interests conflict.

*Note: We aimed in this study to focus on complicated pregnancy from the time of viability, however, the participants pro-actively discussed all aspects of their experiences with the use of ultrasound during pregnancy. The research group made a decision to include these aspects in presenting the results.*

## Methods

### Study design

The study, undertaken as part of the CROCUS-project [11], had a qualitative design involving focus group discussions (FGDs) with midwives working in antenatal and intrapartum care in Victoria, Australia.

### Participants

Participants were recruited from two large hospitals (each with more than 4000 births per year) in Victoria, Australia. The recruitment was organised via the department head at one hospital and a midwifery manager at the other. Both were asked by the research team to provide midwives with information about the study and ask for their participation. We aimed for six to eight midwives in each group and for involvement of midwives with varying experiences of caring for women with both high and low-risk pregnancies. The actual group sizes varied between two and 12 participants depending on work load at the time of each FGD. The recruited participants were female and all registered midwives, except for two who were student midwives. They had varying ages and work experience. One third of the participants had specialised training in performing ultrasound examinations. The characteristics of participants are presented in Table 1.

**Table 1:** Characteristics of midwives participating in focus group discussions (*N* = 37)

Focus group No.	Number of participants	Age, mean (range years)	Work experience as midwife, mean (range years)	Specialised training for ultrasound examinations, n
1	3	44 (31–55)	18 (9–30)	2
2	2	52 (50–54)	29 (26–32)	2
3	12^a^	34 (23–51)	7 (1–28)	0
4	9	40 (25–58)	13 (2–30)	0
5	6	44 (25–58)	14 (1–30)	5
6	5	44 (27–56)	17 (4–34)	3

^a^Including two student midwives

### Data collection procedures

The FGDs were held during the midwives’ normal work shift in separate rooms on the wards, with attendance supported by their manager or department head. Prior to the start of each FGD, the participants were asked to complete a brief questionnaire reporting their age, work experience and if they had specialised training in performing obstetric ultrasound examinations. An interview guide developed for the CROCUS-project was used (Table 2). The FGDs lasted between 35 and 60 min (mean length 45 min), and they were digitally recorded with the consent of participants. Data collection took place during one week in November 2012, and the authors met daily during this week to discuss the outcomes of each FGD and for planning of forthcoming FGDs. By the sixth FGD, it was clear that further data collection was unlikely to provide any new information in relation to the purpose of the study, indicating that data saturation had been reached [12].

**Table 2:** Key domains in the CROCUS interview guide

Key domains
The midwives’ views/experiences of:
• The importance/value of obstetric ultrasound for clinical management of complicated pregnancy.
• The importance of obstetric ultrasound in comparison to other surveillance methods during complicated pregnancy.
• Clinical situations where the interests of maternal and fetal health conflict.
• Whether the woman may be considered to act as an instrument for fetal treatment.
• If/when the fetus can be regarded as a person.
• Situations where the fetus has been regarded as a patient with his/her own interests.
• Their professional role in relation to other occupational groups working with obstetric ultrasound examinations or the outcomes of these examinations.
• Other issues in relation to ethical aspects of the use of obstetric ultrasound.

### Data analysis

All digital recordings were transcribed verbatim and analysed using qualitative content analysis informed by Graneheim and Lundman [13]. This process included reading of interviews to identify emerging topics (KE, and RS), coding all data and subsequently sorting the codes into broad content areas based on assessment of their similarities and differences (KE). The codes were lengthier in order to ensure that the context was not lost in the sorting process. The content areas were then further refined and divided into categories and subcategories (KE, RS). An overall theme emerged during this process. Last, the preliminary results were discussed between all authors until consensus was reached regarding interpretation of findings and labelling of categories and the theme.

### Ethical considerations

Written and verbal informed consent was obtained from all participants, and all participation was voluntary. Ethics approval was obtained from the Faculty of Health Services Human Ethics Committee at La Trobe University in Melbourne (reference FHEC12/135) and the Human Ethics Committees of the two participating hospitals.

### The researchers’ backgrounds

The research group represents both qualitative and quantitative research traditions and various professional disciplines including midwifery, nursing, maternity services and maternal health research, obstetrics and gynecology, behavioral science, public health and epidemiology.

## Results

One overarching theme emerged from the analysis: *Obstetric ultrasound – a routine tool with far-reaching influence.* It was built on three categories: *‘Experiencing pros and cons of ultrasound’*, *‘Viewing ultrasound as a normalised and unquestioned examination’* and *‘Reflecting on the fetus as a person in relation to the pregnant woman’* (Table 3). In presenting the results, the main findings will be summarised, and each category will then be presented with its subcategories, together with representative quotes from participants (italics).

**Table 3:** Theme, categories and sub-categories

Theme	Category	Sub-category
Obstetric ultrasound – a routine tool with far-reaching influence	I. Experiencing pros and cons of ultrasound	Optimising pregnancy outcomes
		Providing choice, reassurance and boding
		Contributing to medicalisation of pregnancy
		Leading to complex decision-making dilemmas and parental anxiety
	II. Viewing ultrasound as a normalised and unquestioned examination	A standard component with diverse meanings
		A fully informed choice?
		Reducing societal tolerance for disability?
	III. Reflecting on the fetus as a person in relation to the pregnant woman	Visualisation technology contributes to personification of the fetus
		The law versus personal views
		Stating maternal rights as the first priority

### Obstetric ultrasound – a routine tool with far-reaching influence

Overall, ultrasound was seen as having many advantages in maternity care; however, it was also seen as contributing to increased medicalisation of pregnancy, to complex and sometimes uncertain decision-making and to parental anxiety. The use of routine ultrasound was described as normalised and unquestioned in health care and in wider society. Midwives were concerned that this impacts negatively on informed consent processes, and at a societal level, to threaten acceptance of human variation and disability. Further, the use of ultrasound was seen to have led to increased ‘personification’ of the fetus, though a variety of views were expressed about when the fetus ‘becomes a person’. Protection of maternal health was the first priority for midwives, but they felt that women often put fetal health interests ahead of their own.

### I. Experiencing pros and cons of ultrasound

There was ambivalence apparent among participants with regard to the role obstetric ultrasound plays in maternity care. The discussions oscillated between pros and cons both from the perspectives of the health care providers and the expectant parents. The participants described themselves in general as confident in undertaking basic and routine scans such as estimating amniotic fluid index, the systolic/diastolic (SD) ratio, fetal presentation, or fetal movements. However, they saw themselves as having a background role in decision-making in relation to obstetric ultrasound; making decisions about obstetric management based on ultrasound results was clearly seen as the doctor’s responsibility, and some midwives saw it as a relief that they did not have to make vital decisions because of concerns about liability.

#### Optimising pregnancy outcomes

From the clinical perspective, ultrasound was seen as an essential tool that had changed pregnancy management. It was said to play a crucial role in monitoring fetuses with in utero problems, such as in the management of intrauterine growth retardation and placental problems, and in the timing of delivery. Ultrasound was also seen as an important screening and diagnostic tool, as abnormalities could be picked up early in pregnancy and adverse outcomes avoided or reduced. For example, by detecting abnormalities such as heart problems, relevant specialist expertise could be prepared for at the birth of the baby and appropriate resources put in place.

*‘They’re [ultrasounds] very good preventative [tools], because we catch a lot of things before it could go really, really wrong.’* (FGD 3)

Some participants also discussed the fact that ultrasound has become an increasingly important tool as overweight and obesity have become more prevalent in pregnant women. It was said to be very difficult clinically to assess adipose women, and ultrasound could in such situations assist the midwives in their assessment. At the same time, the problems with lower image quality in obese women was also raised as a concern, particularly in relation to the risk of missing deviations, primarily fetal heart abnormalities.

*‘BMIs greater than 30…no matter how fantastic your clinical skills are… you cannot assess that.*’ (FGD 3)

#### Providing choice, reassurance and bonding

The participants also saw obstetric ultrasound as highly important in relation to giving expectant parents a choice about whether to continue with the pregnancy or not, in situations when abnormalities were detected. They also discussed that, in the event of a fetal abnormality, some expectant parents could benefit from learning early about the problem, as opposed to going through nine months of pregnancy expecting a healthy baby. Furthermore, ultrasound was perceived to be important for parental reassurance, especially after experiencing previous pregnancy complications or stillbirths.

*‘When they’ve got a poor obstetric history, like if they’ve had a fetal death in utero or a previous stillbirth or things like that, sometimes in the next pregnancy we are doing it [ultrasound examination] more just for maternal anxiety.’* (FGD 3)

Ultrasound’s role in increasing attachment and promoting expectant parents’ bonding to the pregnancy – not least for the partners – was also emphasised.

*‘They [expectant parents] do bond and they love looking at their baby.’* (FGD 4)

#### Contributing to medicalisation of pregnancy

On the other hand, obstetric ultrasound was also pictured as an important contributor to the increasing ‘medicalisation’ of pregnancy. From being something natural and somewhat ‘mysterious’, pregnancy was said to have moved to being highly monitored, controlled and often subjected to a variety of interventions. Alongside other medical advancements, including new reproductive technologies, ultrasound was described as having the potential to undermine normal pregnancy and childbirth processes.

*‘We have certain guidelines, we have to go to the doctors, so of course they’re going to then use medical technology and it just flips over from being a normal pregnancy to being a disease almost.’* (FGD 5)

Although participants also saw ultrasound as important in confirming their clinical findings, an obstetric ultrasound examination was said to be trusted over and above clinical skills, partly because this ‘evidence’ was more concrete or visible. The conflict between aiming to preserve the natural birthing process, but at the same time having to act upon ultrasound findings to optimise pregnancy outcomes was frequently discussed. One situation described was the use of ultrasound for measuring fetal weight. According to the participants, weight estimates via ultrasound were not always accurate, and women were subsequently sometimes induced because of false signs of a small or large baby. The midwives felt that ultrasound sometimes leads to unnecessary interventions including caesarean sections.

*‘[Ultrasound] leads to more intervention also but at the same time we’re probably saving more babies.’* (FGD 1)

#### Leading to complex decision-making dilemmas and parental anxiety

The participants also saw advancements in imaging technology as setting the scene for more complex decision-making dilemmas for both caregivers and expectant parents. Situations often mentioned were when uncertain findings that might represent abnormality were revealed, but no one could be certain about the implications of the findings. Some presented the view that the constantly improving imaging technology would lead to more dilemmas in the future, this because the knowledge of how to interpret findings would not keep up with the technical development.

*‘I think they will… be finding [in the future] more and more things that they just don’t know what it means…’* (FGD 3)

Ultrasound findings falsely indicating that something was wrong were described as having significant consequences in terms of unnecessary worry and anxiety for expectant parents. Evaluating probability figures regarding abnormalities was also described as creating concern and stress for some expectant parents, as this could be difficult to make sense of or grasp.

*‘…you tell parents that there’s something not quite right with the baby’s brain, well it doesn’t matter how you expand that up, it’s going to create a lot of anxiety… and at the end they’ve got a normal baby and we’ve just wrecked that pregnancy really. I see it quite often…’* (FGD 3)

*‘I see the value in it but I also see the stress it can cause when you get a result that it’s like one in 150 or one in 200 chance of an abnormality.’* (FGD 6)

Another example of a complex dilemma resulting from imaging possibilities in combination with medical advances was selective abortion in multiple pregnancies.

*‘I remember there was a woman with triplets and she ended up making the decision to have a selective termination and actually all three died so that would never have been offered 10 years ago… we sort of have the technology to not always make our lives less complicated.’* (FGD 6)

The participants said that they were conscious about how they expressed themselves in relation to ultrasound findings so as not to increase expectant parents’ worries more than necessary. They also described treading very carefully when discussing findings from scans performed by obstetricians or sonographers to prevent giving mixed messages in situations of uncertainty. Some participants presented the view that ultrasound was not to blame for causing anxiety, rather it was an issue related to whether the person doing the scan counselled the woman and her partner appropriately or not.

### II. Viewing ultrasound as a normalised and unquestioned examination

#### A standard component with diverse meanings

The participants talked in different ways about how ultrasound has come to be accepted as a norm in maternity care as well as in the wider society. Ultrasound was perceived as being an unquestioned and integral part of pregnancy management.

*‘It’s become so used and so accepted without necessarily … you just see people … yeah, accepting and loving the technology.’* (FGD 5)

It was also described as being highly valued by expectant parents; the routine ultrasound was an event in pregnancy most looked forward to. Even though some pregnant women were described as ‘poor attenders’ in antenatal care, the midwives claimed that very few would miss out on an obstetric ultrasound examination.

*‘Most of them look forward to it, they see it as the time that they first connect [with] their baby.’* (FGD 6)

The participants’ discussions recurrently suggested that ultrasound had to some extent different meanings for health care providers and expectant parents. While the medical aspect of ultrasound provided the rationale for its use for providers, the midwives experienced that expectant parents greatly valued the psychosocial aspects of ultrasound. This included’ seeing the baby’, finding out the sex, getting pictures and also bringing other family members along to the examination to share the special moment of ‘seeing the baby’ for the first time. Some participants suggested that expectant parents sometimes viewed obstetric ultrasound as ‘entertainment’ rather than a medical examination. This meant also that they were poorly prepared in the event of any adverse finding.

*‘They think they’re going for a scan that’s going to give them nice pictures of their beautiful baby that they can show to everyone.... and find out if it’s a girl or a boy. They don’t think past that point.’* (FGD 3)

#### A fully informed choice?

One of the implications of routine ultrasound being normalised as part of standard maternity care, was said to be the issue of informed consent. The ‘routine’ use of ultrasound in pregnancy meant that most expectant parents would not question having the ultrasound examination, and the midwives commented that the majority of people would not think of having ultrasounds as well as combined screening tests in pregnancy as a choice.

While some perceived that women’s understanding of ultrasound was better today than some years ago, others thought that expectant parents in general were not fully aware of the purpose and the potential outcomes of routine obstetric ultrasound, nor the limitations of the procedure. They also thought that the information provided about the examination was insufficient. This could result in an increased risk for distress if the ultrasound provided signs or evidence of abnormality, or if at childbirth the baby was born with an abnormality that had not been detected by ultrasound. The participants also believed that it was harder for women these days to decline routine examinations as they have become the norm in pregnancy management. Furthermore, some thought that women who declined ultrasounds risked being perceived as irresponsible by the hospital, as well as the community.

*‘Do women get a choice though? Usually it’s you get the slip to go and get your ultrasound.’* (FGD 2)

*‘A lot of them think that they’re going there, yay we get to see the baby and we get to find out the sex. They don’t actually understand the significance of the scan and what we’re looking for. And then if something does turn up, then they’re completely devastated because nobody told me you were looking for that, I thought we were going to find out the sex of the baby.’* (FGD 2)

It was some midwives’ experience that women who actively declined routine obstetric ultrasound were in general better informed than those who accepted it without a second thought, and that their decisions were more likely based on truly informed consent. According to the participants, among women who declined routine ultrasound were those who felt they would not do anything with the information, those who did not want to face difficult decisions, and those women who thought that the ultrasound could be harmful to the fetus. Although some participants depicted these women as a bit ‘alternative’, they expressed at the same time their understanding and respect for these women.

*‘The ones that choose not to are far more informed than the ones that choose to – because you have to go against the system.’* (FGD 5)

Some midwives believed that women from lower socioeconomic backgrounds in general had a more naive understanding about the purpose of the routine ultrasound (i.e., seeing the sex), compared with those from higher socioeconomic groups who were considered to be more capable of searching out information.

#### Reducing societal tolerance for disability?

The participants also reflected on ultrasounds’ impact on the wider society in relation to being a normalised and unquestioned tool. Some participants thought that it had become less acceptable in the community to have a child with a disability because of the possibilities of detecting abnormalities via ultrasound and through other available screening and diagnostic procedures. They discussed the reduced tolerance for human deviations that put pressure on pregnant women to conform, i.e., to undergo screening tests and act upon adverse findings by for example requesting an induced abortion. One participant even commented that this was akin to ‘genetic cleansing’:

*I was just thinking of how I guess in society now there’s a bit of genetic cleansing… So you know like Down syndrome children, you know 30, 40 years ago it was Down syndrome children in the community. But it’s very difficult now for people to … because they’re not seen…* (FGD 2)

### III. Reflecting on the fetus as a person in relation to the pregnant woman

#### Visualisation technology contributes to personification of the fetus

The participants discussed how society’s views of the fetus had changed because of the use of ultrasound in pregnancy. The visualisation of the fetus was felt to have contributed to the fetus being perceived as a ‘baby’ at an early gestational age. The pregnancy and the ‘baby’ were also said to become more real when the human features were visualised.

*‘We have the scans and we can see these little babies from a very early age. It’s very different than the concept of a baby that’s actually inside a mother’s tummy… now there are 3D photos, you see their little noses… that in itself has made the baby kind of human …’* (FGD 6)

The participants also commented that expectant parents often named their fetus as soon as they found out about the sex, and that the fetus thereafter was called their ‘baby’ or ‘child’, contributing to ‘personification’ of the fetus long before viability.

*‘A lot of people who are not the pregnant woman want to be able to see the fetus before it’s born and I think that… with determining sex and naming the baby and getting the pink wardrobe or the blue wardrobe or whatever really contribute to the… to society’s feeling that this is a baby and not a fetus.’* (FGD 1)

This early ‘personification’ was said to have implications in pregnancies with poor outcomes, as it was perceived more difficult for women and their partners to deal with fetal loss if the fetus was viewed as a baby at an early stage in pregnancy.

#### The law versus personal views

Views about when the fetus can be regarded as a ‘person’ were said to be dependent on whose perspective was being considered: expectant mothers and fathers, health professionals or the law. While expectant parents were seen as often perceiving the fetus as a person at a fairly early stage in pregnancy, the law does not recognise the fetus as a person until it is born.

*‘If it’s unborn [the fetus], it doesn’t really have any rights.’* (FGD 5)

The midwives themselves had differing personal views about if or when the fetus could be regarded as a person, although they underlined that the legal definition guided their work. Some thought that the fetus could be regarded as a person from conception; others at later gestational age, and some that the fetus became a person at birth. Many factors were said to influence personal views, including religious and spiritual factors.

*‘I’d say I think the fetus is from around 24 weeks, that once the fetus is viable then I think that it’s a person.’* (FGD 4)

Although the law stood in conflict at times with the participants’ personal opinions, it was also described as assisting in protecting their own emotional wellbeing in difficult situations generated by ultrasound findings.

*‘I think we cover ourselves emotionally and spiritually as well by following the … the legal guidelines.’* (FGD 5)

Some raised the concept of the fetus as a patient, and independent of gestation, that the fetus was viewed as a patient as soon as it was subject to intrauterine treatment with the consent of the pregnant woman.

*‘We may not register them in the hospital but I think they are a patient as well.’* (FGD 4)

#### Stating maternal rights as the first priority

The participants unanimously said that the health of the pregnant woman was the number one priority in pregnancy management. It was clearly stated that the fetus did not have any rights prior to birth. This meant that decisions were in the hands of the pregnant woman at all times, even in the situation where the health interest of the pregnant woman was in conflict with the health interest of the fetus. The participants emphasised that the pregnant woman was thus in charge of all decisions related to fetal interventions.

*‘The health of the mother is the first priority. And then obviously the health of the baby comes a very close second, but priority would be always the mother over the baby if there was anything major happening.’* (FGD 5)

However, it was described as fairly common that women would care more about potential problems for the fetus than they cared about the potential problems for themselves, even if their own health was at severe risk. The midwives described themselves as uncomfortable with seeing a woman’s health deteriorate greatly because of her decision to ‘sacrifice’ her own health in favour of fetal health.

*‘She was a woman who struck me as like she was almost willing to … she was like I’ll carry this pregnancy ‘til it kills me if it’s better for the baby*.’(FGD1)

A few participants raised paternal rights as an issue, but concluded that paternal rights really did not exist in pregnancy related decision-making.

## Discussion

The present study has demonstrated that the use of ultrasound during pregnancy has had far reaching influence. The results reflect how normalised ultrasound has become in Australia and how it has shaped the way in which the pregnant woman and the fetus are viewed among midwives. Moreover, the study illuminates some unforeseen consequences of ultrasound use for pregnancy decision-making.

The first category of results illuminates midwives’ perspectives on the pros and cons of ultrasound in pregnancy care. Although the results highlight many benefits, ultrasound was also depicted as contributing to increased medicalisation of pregnancy, this because it was seen as sometimes being the beginning of an altered pathway of care. The aspects of increasing medicalisation of pregnancy and childbirth as described in this study resonate with previous reports about increasing use of interventions and reduced ‘normality’ of childbirth globally [14, 15]. However, ultrasound’s role in the complex range of factors that lie behind this trend is yet to be determined. Increasing medicalisation of childbirth is undeniably also an issue in Australia. The country has seen induction of labour increase steadily over recent decades, an increase that has not been accompanied by improved neonatal and maternal outcomes [16]. Furthermore, the overall caesarean section rate reached 32.4 % in 2012, with a strikingly high rate of 43.6 % in private hospitals [17]. One of the core tasks of the midwife is to promote and support normal birth [18]. The concerns the participants raised may thus be seen as an expected response to the increasing medicalisation of Australian maternity care. Midwives may in some situations be limited in the extent to which they can exercise their expertise, especially in contexts where the fear of liability is frequently present, something that was also mentioned by the participants. One important example is given by a previous study of Australian midwives who worked at a tertiary maternal referral centre which was the subject of an extensive external review of obstetric services as well as a number of legal proceedings. These midwives described acquiescing to an increasingly medicalised defensive practice and they felt unable to take full advantage of their skills to provide a supportive, nurturing atmosphere where they could support normal birth [19]. Barriers for optimal care may exist at several levels of the health care system, including the level of the patient, the professionals, the social context, the organisational context and the economic and political context [20]. It therefore seems important to identify, address and discuss potential barriers for optimal maternity care from a multi-level perspective in efforts to curb unnecessary medicalisation of pregnancy. Midwives views and opinions play an essential role in these discussions.

Other drawbacks of ultrasound as discussed by the participants included parental anxiety that can result in the event of adverse ultrasound findings, and where findings are uncertain, leading to complex decision making. These results are consistent with previous studies showing that pregnant women can feel anxiety, distress, or disappointment in the event of adverse ultrasound findings, particularly if they lack information about the purpose of the ultrasound, and what the outcomes of a scan can be [9, 10]. On the other hand, the vast majority of ultrasound examinations provide reassurance for expectant parents that everything is fine with the current pregnancy [10]. Previous studies have also indicated that the examination may positively influence maternal-fetal attachment and bonding [21, 22], and may also positively influence expectant fathers’ bonding and feelings towards their fetus [23], something which was also emphasised by our study participants.

The second category of results illuminates the view that ultrasound has become a normalised and unquestioned examination in maternity care. Given its many benefits in obstetric management and its role in optimising pregnancy outcomes in high risk pregnancies [24], and its vast popularity among pregnant women and expectant fathers [9, 10, 25], this development is not surprising. However, the participants provided some interesting thoughts and perspectives on the implications of this ‘normalisation’. First, they questioned whether expectant parents were able to fully exercise their rights of autonomy in relation to decisions about routine pregnancy ultrasound, as well as other prenatal screening tests, as this ‘normalisation’ may mean that these interventions are perceived to be standard and compulsory parts of pregnancy care.

Our results seem consistent with previous research indicating that information about routine ultrasound is often insufficient for women to make an informed decision [26]. Others have suggested that one reason for insufficient information may be that procedures that have become routine and are no longer experienced as new by health professionals may be perceived as not needing so much explanation [9], which our results also indicated. Other reasons may include the fact that ultrasound is so appealing to expectant parents that attention is not paid to detailed aspects of the examinations [9]. Women who are poorly informed about obstetric ultrasound may be put in a very much unwanted situation of having to make decisions about their unborn child; a situation that entails balancing the interest of the fetus, the family and personal values and circumstances [27, 28]. Thus, in efforts to improve informed consent in relation to ultrasound examinations in pregnancy, it seems important first to determine the underlying causes for women being inadequately informed, and equipped with the results, develop better information strategies.

The participants also raised concerns over the possibility that pregnant women may feel pressured to conform to expectations of undergoing ultrasound examinations, and to act upon adverse ultrasound findings. These results are consistent with previous studies that have shown that women may perceive screening tests during pregnancy as ‘a standard component of routine care’ rather than as a choice, something they may feel subtle or overt pressure to accept [29–31]. One important point made by participants was that by declining ultrasounds, and thus not complying with the ‘default’ option and the prevailing norm, women risked being perceived as ‘alternative’ or ‘irresponsible’. The study participants’ thoughts that expectant parents’ may feel pressured to conform to expectations of how to act upon adverse ultrasound findings also have strong support in previous quantitative and qualitative research and narratives [31–36]. For example, in a recent study of parents who decided to continue their pregnancy despite a prenatal diagnosis of trisomy 13 or trisomy 18, 61 % reported feeling pressure from health care providers to terminate the pregnancy [33]. Many factors influence parents’ decision to continue a pregnancy despite identification of chromosomal aberrations or other fetal anomalies, including personal values and beliefs [33, 37, 38]. Considering that these parents may have positive and enriching experiences regardless of the child’s disability or shortened lifespan [33, 34], it is imperative for health professionals to provide non-directive counselling when fetal abnormalities are detected through routine ultrasound. Further studies exploring expectant parents’ experiences of health professional’s attitudes, approaches and information in these contexts are needed. Forthcoming studies within the CROCUS-project will involve expectant parents’ perspectives on these issues.

The study participants discussed issues related to the use of ultrasound not only from the perspective of the parents and the maternity care givers, they also reflected on the impact of obstetric ultrasound on the wider society in terms of reduced tolerance for human deviations and disability. Others have also touched upon the concerns our participants expressed. For example, in an interview study with women who were in the process of deciding about prenatal screening, it was found that some women expressed similar thoughts to our study participants saying that ‘only perfect children may be born in the future’. That is to say, today’s possibilities for influencing pregnancy outcomes drive the society in the direction of expecting only ‘perfect’ babies to be born to the world, and thus, reducing the society’s acceptance for human deviations [27]. The study was undertaken in the Netherlands where routine screening examination was not yet a part of prenatal care. Others have also discussed the risk that efforts to eliminate congenital disability may promote a cult of perfectionism in the society, and ultimately, increased discrimination against people with disabilities [39].

The third category of results illuminates the role ultrasound plays in ‘personification’ of the fetus, and its role in relation to the pregnant woman. The use of and advances in ultrasound were seen to have led to the fetus coming to be regarded as a ‘baby’ or a ‘person’ at an early stage in pregnancy. These results resonate with previous discussions of the influence of imaging technology on ‘personification’ of the fetus, and some argue that the construction of a fetal ‘personhood’ may have adverse consequences for women’s reproductive freedom [40]. One of these consequences is that attention is increasingly being directed to the situation of the fetus, which in turn entails a risk that the pregnant woman loses her central role in pregnancy. Increased ‘personification’ of the fetus has also raised the issue of fetal rights, and imaging technology has been a powerful tool for anti-abortionists [40, 41]. Fetal rights prior to birth would undeniably lead to complex medical, ethical and legal situations in obstetric care; it would also violate women’s reproductive freedom [40]. However, the participants in this study thought that the interest of the pregnant woman should be prioritised in all situations of maternal and fetal health conflicts. This finding to some extent seem inconsistent with a previous Australian study showing that doctors in general believed the needs of the woman have to be overridden for the safety of the fetus in some situations, while midwives overall were neutral to this statement [42]. The results that pregnant women often would care more about potential problems for the fetus than they cared about potential problems that could affect themselves are in line with those of one of our previous studies involving Australian obstetricians. This previous study outlined that pregnancy management was particularly difficult in circumstances when the risk to the pregnant woman was high while at the same time fetal benefit would be unlikely [43].

Reports on the development of maternity care in Australia suggest that midwives exist in a paradigm shift where normal pregnancy and childbirth is given less room in favour of more medicalised care, particularly in private hospitals [44]. It is clear from this study that midwives may have paradoxical feelings about this development. Midwives may be particularly exposed to the outcomes of obstetric ultrasound examinations as they are the professional group working closest to pregnant women and their partners in maternity care. They are the ones who often support women after they receive adverse information from an ultrasound examination. This means that continuing guidance, education and support are needed to ensure they can provide appropriate support to pregnant women and their partners in these situations. This study indicates that pregnant women/expectant parents may have varying needs for information about ultrasound examinations in pregnancy. It is therefore important that health care providers put emphasis on individualising information in all encounters with expectant parents.

### Strengths and limitations

One strength of this study is that the use of FGDs generated rich discussions of views and experiences of practising midwives (*n* = 37), with varying characteristics in relation to age work experience and setting (two different hospitals). Another strength is that the researchers’ various backgrounds and experiences contributed diversity of perspective in the process of analysis and reporting, something we believe increases the trustworthiness of the study results. One limitation of the data collection method lies in possible issues of conformity; during FGDs participants might have withheld issues they may have talked about more easily in the context of an individual interview [45]. Another limitation is that some of the FGDs exceeded the intended group size, while two groups only had 2 and 3 participants, respectively. Furthermore, we aimed in this study to focus on complicated pregnancy from the time of viability. However, on their own initiative the participants raised other aspects related to the use of ultrasound that might not have been explored in sufficient depth, for example the use of ultrasound for screening purposes in early pregnancy. In interpreting the results from this study, it is important to note that our study participants were recruited from tertiary referral hospitals with health care for both high-risk and low-risk pregnancies. Thus, they were working in highly specialised and intervention-rich environments.

## Conclusions

The results reflect the significant influence ultrasound has had in maternity care and highlights ethical and professional challenges that midwives face in their daily working lives concerning its use. Further discussion about the use of ultrasound is needed, both among health professionals and in the community, in order to protect women’s rights to informed decision-making and autonomy in pregnancy and childbirth and to curb unnecessary medicalisation of pregnancy. Midwives’ experiences and views play an essential role in such discussions.
